# Novel detection of provenance in the illegal wildlife trade using elemental data

**DOI:** 10.1038/s41598-018-33786-0

**Published:** 2018-10-18

**Authors:** Kate J. Brandis, Phoebe J. B. Meagher, Lydia J. Tong, Michelle Shaw, Debashish Mazumder, Patricia Gadd, Daniel Ramp

**Affiliations:** 1 Centre for Ecosystem Science, School of Biological, Earth and Environmental Sciences, University of New South Wales, Kensington, 2052 NSW Australia; 2 Taronga Wildlife Hospital, Taronga Conservation Society Australia, Mosman, 2088 NSW Australia; 3 Australian Nuclear Science Technology Organisation, Lucas Heights, 2234 NSW Australia; 4 Centre for Compassionate Conservation, University of Technology Sydney, Ultimo, 2007 NSW Australia

## Abstract

Despite being the fourth largest criminal market in the world, no forensic tools have been sufficiently developed to accurately determine the legal status of seized animals and their parts. Although legal trading is permissible for farmed or captive-bred animals, many animals are illegally removed from the wild and laundered by masquerading them as captive bred. Here we present high-resolution x-ray fluorescence (XRF) as a non-invasive and cost-effective tool for forensic classification. We tested the efficacy of this technique by using machine learning on a training set of zoo specimens and wild-caught individuals of short-beaked echidnas (*Tachyglossus aculeatus*), a small insectivorous monotreme in Australia. XRF outperformed stable isotope analysis (δ^13^*C*, δ^15^*N*), reducing overall classification error below 4%. XRF has the added advantage of providing samples every 200 μm on a single quill, enabling 100% classification accuracy by taking the consensus of votes per quill. This accurate and cost-effective forensic technique could provide a much needed *in situ* solution for combating the illegal laundering of wildlife, and conversely, assist with certification of legally bred animals.

## Introduction

The international illegal wildlife trade (IWT) is the fourth largest criminal market in the world, worth between USD$7-23 billion annually^1^. Hundreds of millions of plants and animals are imported annually into the USA and Europe, used for medicine, food, pets, or fashion. The cost of IWT to biodiversity and species conservation is considerable^2–4^, as are costs to the individuals being caught and traded. Impacts occur during capture, both to the individuals being caught and to inhabitants of the wider ecosystem, and post-capture, including ongoing welfare harms to individuals^5^ and the potential spread of zoonotic and agricultural diseases^6,7^. While many animals are traded as body parts, the live pet trade is an increasing driver of biodiversity loss, particularly in the Asia-Pacific region^7–9^. Since 1973, the Convention on International Trade in Endangered Species of Wild Fauna and Flora (CITES) has provided regulation of international trade of over 5,800 fauna species, covering endangered species, species subject to exploitation (e.g. exotic pets), and nationally protected species. Successful enforcement of the Convention is generally regarded as critical for combating IWT, although schemes for disincentivising trade and stimulating community behaviour change are also important^10,11^. However, enforcement falls to member countries and is typically carried out by customs officers. Problematically, although reports on wildlife seizures provide estimates on trade flows^12^, fraudulence is widespread, with much larger volumes seized than could possibly be legally obtained^13^.

Under CITES agreements, certified breeding farms are permissible for some species^3,14–16^. Breeding farms are approved to ostensibly reduce pressure from illegal poaching on wild populations and to assist in biodiversity conservation^17^. Their good intent is often thwarted by the establishment of unlicensed illegal breeding farms^18^ and the illegal sourcing of founder stock from the wild^19^. Furthermore, there are serious concerns that wildlife breeding farms are being used to launder illegally caught wildlife^16,17,20,21^. For example, at least 80% of green pythons (*Morelia viridis*) exported from Indonesia are suspected to be illegally wild-caught^17^. Likewise, illegal laundering of crocodile lizards (*Shinisaurus crocodilurus*), listed as endangered on the IUCN Red List, has been implicated in its decline in Vietnam due to unsustainable exploitation^22^. To combat this, much emphasis has been placed on tracking illegal trade networks^23,24^ and in developing forensic tools to determine the origins of species being traded^22,25^. However, stemming the illegal trade is challenged because existing forensic tools are either inaccurate, inaccessible, or too expensive, making it difficult to confidently determine specimen identity, geographic origin, or legal status.

Forensic tools have primarily focused on the determination of species identity, necessary to prevent the laundering of prohibited species under the name of similar but legally-tradable species^26^. Genetic techniques, like DNA metabarcoding, are suited to this application and have been used to successfully classify specimens^26,27^. A further focus is determining the geographic origins of specimens, particularly for species with large geographic ranges, like pangolin, reptiles, and birds^13,25,28^. Efforts to forensically test for geographic origin have centred on analysis of stable isotopes^29,30^, relying on intrinsic tissue signatures fractionated from diets during assimilation^31–33^. Keratinous tissue such as hair, feathers, scales, and nails, are metabolically inert following synthesis and so maintain an isotopic record reflecting the time and place where the tissue was synthesized, dependent upon temporal and spatial feeding habitats of different species and on tissue specific growth and moulting rates^29^. Importantly, isotopic signatures can be used to detect laundering in IWT by discriminating between captive-bred and wild-caught animals. Some success has been had with reptiles in Vietnam^22^ and with fish in China, Australia, Malaysia, and Indonesia^34^. However, both stable isotopes and genetic testing perform best for geographically isolated species with small home ranges or with restricted ecological niches^22,25^. Furthermore, both tools are expensive, require considerable sample preparation, and cannot be conducted *in situ*, limiting accessibility and versatility.

The lack of a validated, rigorous, and cost-effective forensic method to identify laundered wildlife is a substantial impediment to the enforcement of regulations and laws needed to protect threatened species and wild populations. Here we propose the use of a novel technique to overcome the inherent limitations of existing forensic approaches for specimens with wider geographical ranges and/or more diverse feeding strategies. High resolution x-ray fluorescence (XRF) is a non-destructive analysis tool that measures a wide range of elemental abundances present in samples^35,36^. High resolution XRF has found prominence in its application for elemental analysis of sediment cores and in dendrochronology to determine temporal and spatial variability of samples^37,38^, but can also be used to provide information on geographic location and on diet^39^. For example, elemental analysis has recently been used to determine country of origin for aquaculture shrimp^40^. Despite being readily available, non-destructive, and cost-effective, no previous studies have tested the efficacy of this method as a forensic tool for combating IWT.

As a test case, we chose to employ XRF to discriminate between captive-bred and wild-caught specimens of a species that is highly desired in the international pet trade and which has accessible wild populations and zoo collections, the short-beaked echidna (*Tachyglossus aculeatus*). Short-beaked echidnas are native to Australia, Indonesia, New Guinea, and Papua New Guinea, and can be legally traded from Indonesia if they are accompanied by a permit declaring them as captive-bred. However, echidnas are notoriously difficult to breed in captivity and the survival rate of captive born juveniles is poor. In the forty years preceding 2015, fewer than 20 are known to have been born in captivity and successfully raised in zoos in Australia. Despite this, 25 short-beaked echidnas were seized or traded as captive-bred on Indonesian trade routes between July 2011 and July 2012 alone. Question marks over the legality of seized specimens remain unanswered, stymying enforcement by the lack of evidence proving that specimens were wild-caught and prohibiting certification if they were in fact captive-bred. In this study we utilised both high resolution XRF and stable isotope analysis to classify samples taken from a reference set of echidnas of known origins (either captive-bred or wild-caught). Our goal was to provide an assessment of the merit of adopting elemental analysis as a forensic tool in IWT enforcement.

## Results

### Elements

**Figure 1:**
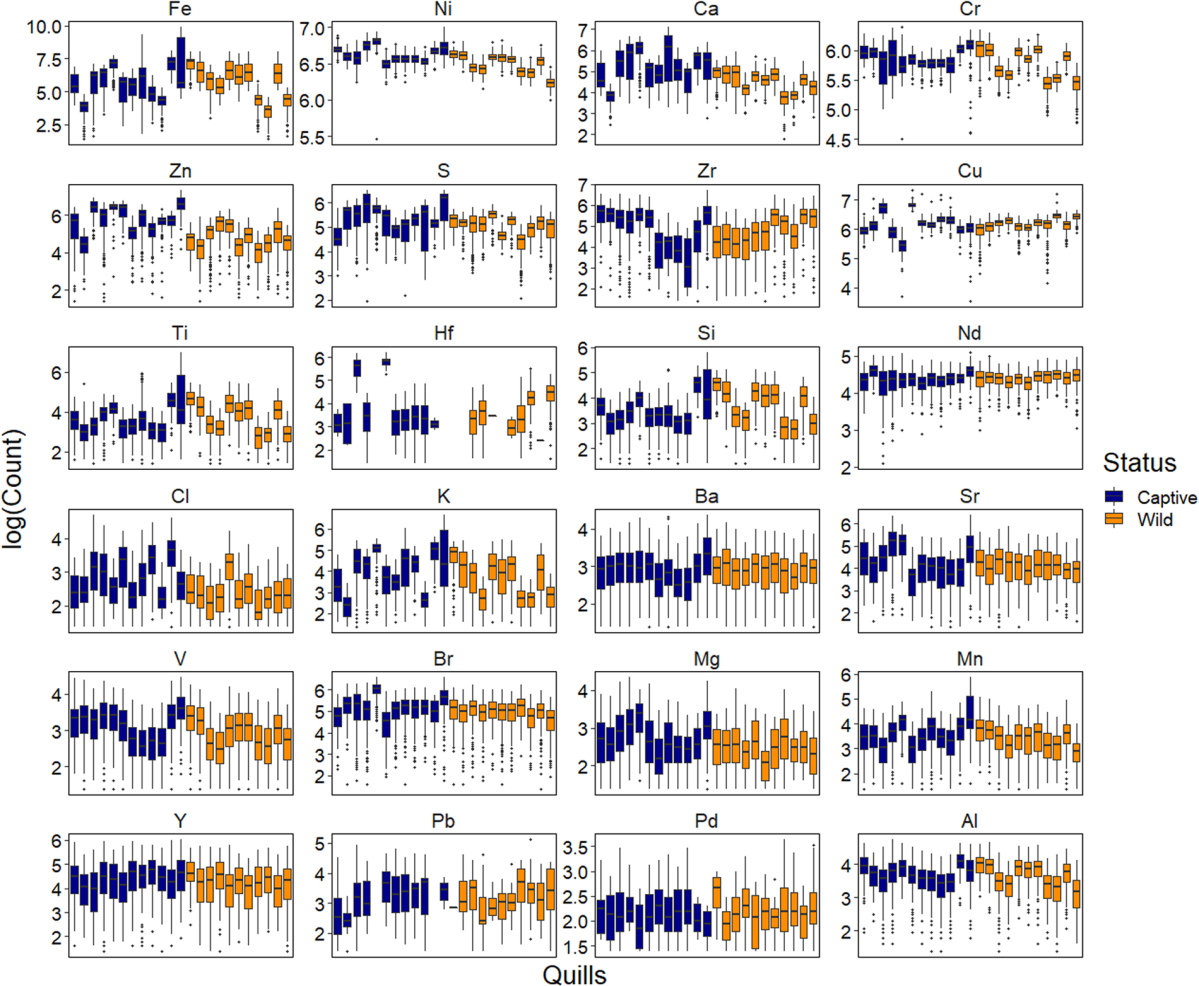
Logged counts of 24 different elements detected at 200-μm intervals on captive-bred (blue) and wild-caught (orange) echidna quills using high resolution x-ray fluorescence, ordered left to right (C1-12 then W1-W11). Elements are ordered from top-left to bottom-right by ranked predictive importance determined using machine learning.

As echidna quills differed in length, the number of XRF scans, spaced every 200 μm, ranged between 94 and 342. Quill lengths did not differ significantly between wild and captive echidnas (*p* = 0.4924) and the median number of samples per quill was 192 for both groups. Univariate testing of elemental differences between captive and wild echidna quills indicated that five of the 24 elements were significantly different, controlling for nesting within each quill (Table 1). In each of the five cases, elemental counts of Calcium (Ca), Magnesium (Mg), Nickel (Ni), Sulphur (S), and Zinc (Zn) were significantly lower in wild caught echidna quills. Variation in counts along each quill were marked, contributing to the lack of discriminatory power among many of the elements when analysed univariately (Fig. 1).

**Table 1:** Univariate generalized linear mixed models fit by maximum likelihood using a negative binomial distribution to account for overdispersion of elemental counts.

Element	Variable	Estimate	SE	*Z*-value	*P*-value
Al	Status	−0.074	0.107	−0.69	0.491
Ba	Status	0.096	0.123	0.781	0.435
Br	Status	−0.149	0.149	−1	0.318
Ca	Status	−0.758	0.222	−3.42	<*0*.*001*
Cl	Status	−0.605	0.34	−1.778	0.0753
Cr	Status	−0.108	0.073	−1.49	0.137
Cu	Status	0.014	0.114	0.12	0.905
Fe	Status	−0.108	0.512	−0.211	0.833
Hf	Status	−1.407	1.843	−0.764	0.445
K	Status	−0.323	0.4	−0.807	0.419
Mg	Status	−0.766	0.279	−2.743	*0*.*006*
Mn	Status	−0.171	0.246	−0.696	0.486
Nd	Status	0.012	0.035	0.35	0.726
Ni	Status	−0.138	0.044	−3.11	*0*.*002*
Pb	Status	0.645	0.666	0.969	0.332
Pd	Status	0.29	0.362	0.801	0.423
S	Status	−0.32	0.14	−2.28	*0*.*023*
Si	Status	0.165	0.264	0.626	0.531
Sr	Status	−0.276	0.354	−0.778	0.437
Ti	Status	0.028	0.316	0.087	0.931
V	Status	−0.304	0.242	−1.257	0.209
Y	Status	−0.241	0.206	−1.172	0.241
Zn	Status	−1.225	0.27	−4.543	<*0*.*001*
Zr	Status	0.231	0.586	0.394	0.693

Each element was modelled separately to test for differences in wild samples from captive samples (Status), where samples were nested within each quill. Coefficient estimates and their standard error are presented along with *Z* and *P* values. Those tests significant at the 0.05 level are italicised.

### Stable isotopes

**Figure 2:**
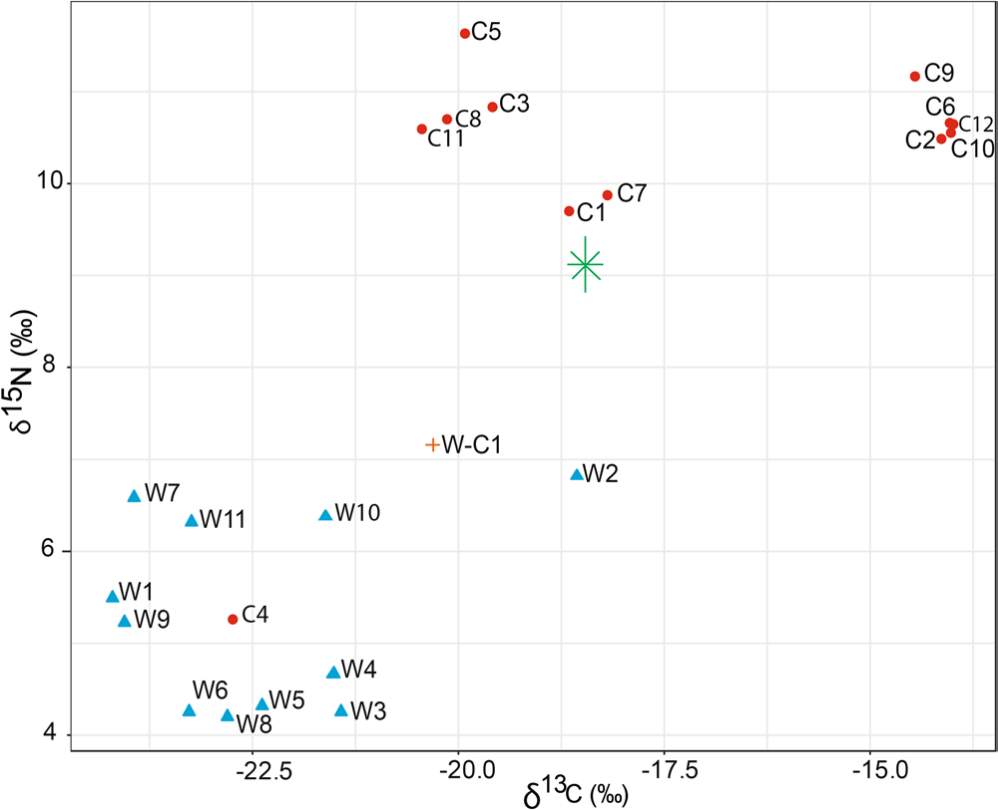
Stable isotope plot of short-beaked echidna quills showing δ^13^C versus δ^15^N. Points represent quills from captive echidnas (red circles), wild echidnas (blue triangles), a wild-captive echidna (orange plus), and the captive food source (green star).

Captive echidna quills had significantly higher values of δ^13^C (*p* = 0.001) and δ^15^N (*p* < 0.001) than wild echidnas (Welch Two Sample t-test). For δ^13^C, values for wild echidnas ranged from −24.2 to −18.56‰ ($\bar{x}$ = −22.46 ± 1.64‰) and from −23.28 to −13.98‰ ($\bar{x}$ = −18.29 ± 3.54‰) for captive echidnas). For δ^15^N, values for wild echidnas ranged from 4.20 to 7.16‰ ($\bar{x}$ = 5.32 ± 1.05‰) and from 7.20 to 11.62‰ ($\bar{x}$ = 9.59 ± 2.14‰) for captive echidnas. Isotopic signatures separated captive-bred quills from wild-caught quills well, except for captive quill 4 (Fig. 2). The quill from a wild-caught echidna that was subsequently in captivity for 5 months (W-C1) was closer in signature to wild echidnas than captive ones.

### Classification

**Figure 3:**
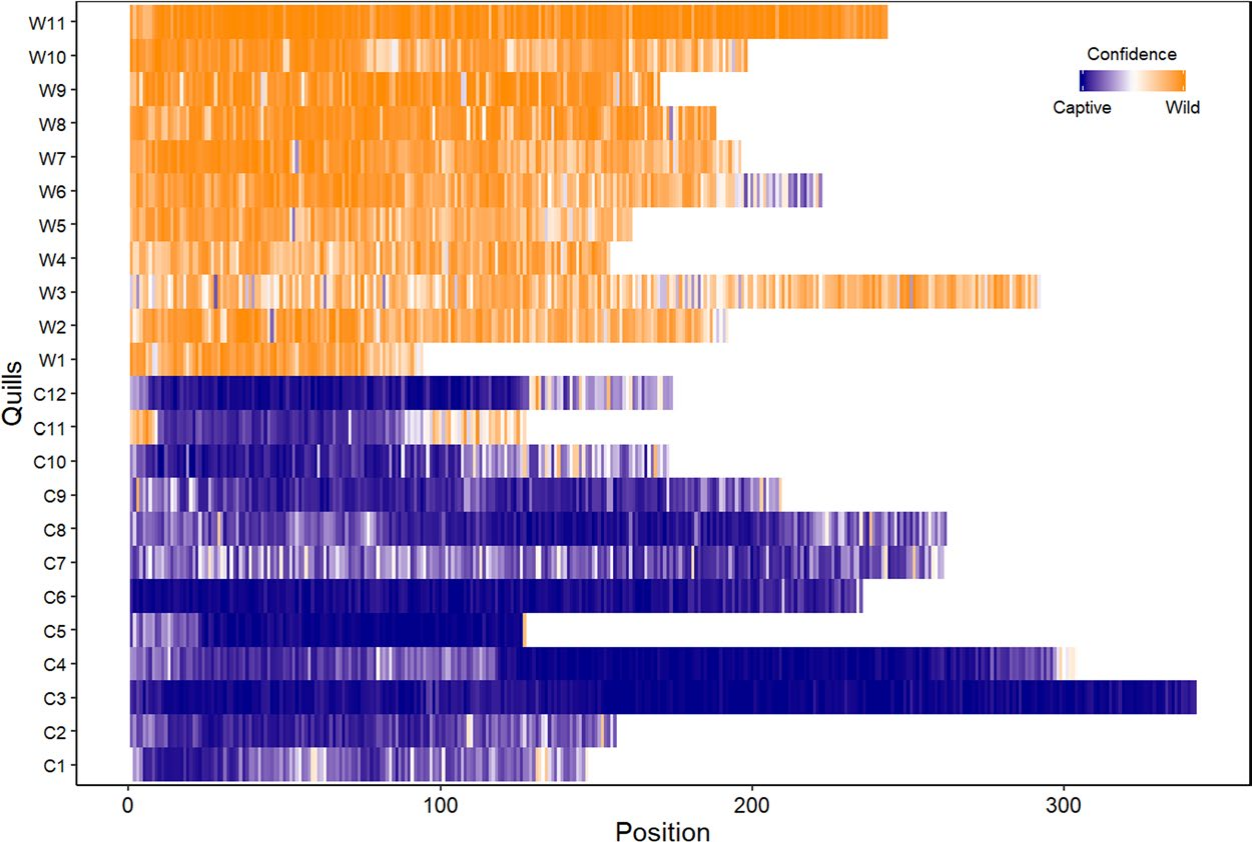
Predicted classification of samples taken from 12 captive quills (C1-C12) and 11 wild quills (W1-W11) using machine learning on 24 elements quantified with high-resolution x-ray florescence. Shading represents the proportion of classification trees supporting the predicted status of wild-caught every 200 microns (voting confidence), where dark blue confidence values indicate strong support for captive-bred and dark orange confidence values indicate strong support for wild-caught. Position 1 represents the tip of the quill (i.e. the oldest sample), with the number of positions determined by quill length.

Machine learning using both elemental and isotopic data could accurately classify quills as either wild or captive (Table 2). Elemental data provided the greatest precision, where only 185 misclassification errors from 4,627 sampling positions across the 23 quills were predicted (3.99%), compared with 2 out of 23 misclassification errors predicted using isotopic data (8.69%). Prediction errors were significantly higher for captive quills than wild quills (t-value = −5.661, *p* < 0.001). At the quill level (Fig. 3), C11 had the highest predictive uncertainty with 27.5% of samples along the quill having more than 50% of trees supporting misclassification, while the next most similar quills with predictive uncertainty were W3 and W6 (with 11.3% and 9.0% of samples supporting misclassification respectively). By aggregating classification predictions from samples along each quill (Fig. 3), consensus among sample classifications predicted quill status 100% of the time. By comparison, classification accuracy based on isotopic data was lower, with one captive quill misclassified as wild (8.33%) and one wild quill was misclassified as captive (9.09%).

**Table 2:** Predicted classification of captive-bred or wild-caught short-beaked echidna quills derived using elements and stable isotopes.

Data source	Status	Captive	Wild	Mean Error (%)
Elemental	Captive	12	0	5.44 ± 2.21
	Wild	0	11	3.32 ± 1.07
Isotopic	Captive	11	1	8.33
	Wild	1	10	9.09

Numbers represent quill classifications based on machine learning conducted on 12 captive and 11 wild quills. Mean error is expressed as the percentage mean number ± standard error of misclassified samples every 200 μm per quill for elemental data, and the percentage mean number of misclassified quills for isotopic data.

Ranking predictive importance using mean decrease in accuracy identified the top five ranked elements for distinguishing captive-bred from wild-caught echidnas as nickel (60.5), iron (57.9), calcium (56.6), chromium (43.4), and zinc (38.5). Partial dependencies of predictor variable influences illustrated that counts were indicative of captive quills when above 600 for nickel, above 200 for calcium, above 300 for chromium, and above 250 for zinc. An absence of iron or very low iron counts were indicative of captive-bred echidnas, while iron counts above 5,000 were indicative of captivity for some echidnas. For the isotopic random forest model, mean decrease in accuracy was higher for δ^15^N (22.9) than for δ^13^C (12.6). Partial dependencies of predictor variable influence illustrated that values above 8‰ δ^15^N and above −16‰ δ^13^C were indicative of captive echidna quills.

## Discussion

**Figure 4:**
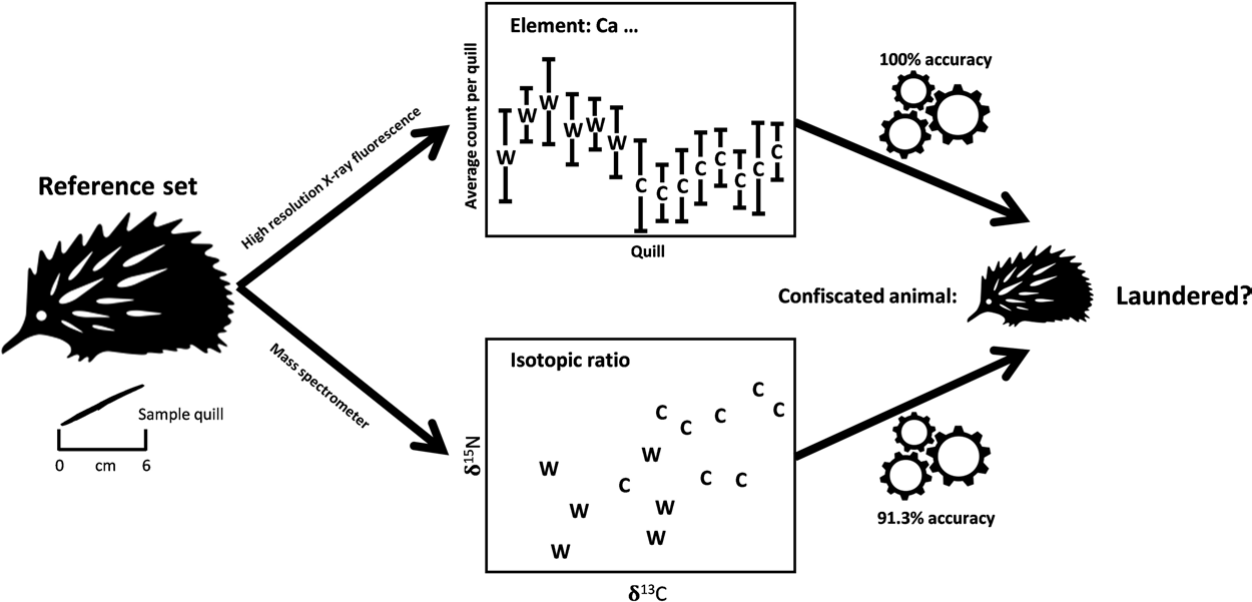
Conceptual model of the forensic approach to detecting illegal laundering of wildlife specimens in the illegal wildlife trade, using elemental and/or isotopic data. Quills from a reference set of echidnas of known origin (i.e. captive bred or wild caught) are non-destructively scanned using high resolution XRF, sampling every 200 μm, then destructively sampled for analysis in a mass spectrometer. Counts for 24 elements are collated and classification accuracy determined by building a machine learning model. Isotopic data are similarly classified, and both models are used to predict the status of confiscated echidnas of unknown origin.

Our study demonstrated the potential utility of high-resolution x-ray fluorescence for distinguishing between captive and wild short-beaked echidnas with a high level of accuracy (Fig. 4). Scans every 200 μm for 24 elements correctly classified samples from echidna quills 95.9% of the time. Furthermore, by taking multiple scans per quill to reflect temporal variation in diet as each quill is developed, classification by consensus was 100% accurate. We validated our findings by confirming that δ^13^C and δ^15^N stable isotopes are useful discriminators between captive and wild individuals, with a classification accuracy of 91.3%.

The utility of ^13^C and ^15^N isotopic data for determining geographic origin is typically restricted to species with narrow dietary niches and restricted home ranges, like echidnas. Species with diverse diets and large home ranges would be expected to exhibit larger variability in δ^13^C and δ^15^N ratios, reducing the discriminatory power to differentiate between groups as potential overlap between diets may occur. This issue does not appear to apply to elemental data because of the large array of elements for which data are collected and the specific locational information that elemental data represent^41^. Rather than reducing classification accuracy, variation in elemental signatures drives predictive performance. High resolution elemental scans along the quill provide a comprehensive analysis of temporal changes that reflect both dietary changes and changes in metabolism during periods of torpor, medical treatment (administering of medicinal drugs to captive animals), sexual development stage, and pregnancy/lactation in females^41^. Considerable variability in elemental abundances were observed in captive echidna quills (Fig. 1), likely due to the large range of ingredient sources with potentially global origins that comprise the captive diet. In contrast, wild echidnas experience less elemental variation in their environment because they have relatively small home ranges and restricted dietary sources, particularly as their primary dietary sources, termites and ants, are highly localized and do not travel large distances. While zoo diets aim to meet nutritional needs of the animal^42^, we showed that the diets of the two groups differed both elementally and isotopically. Captive diets were enriched in δ^15^N, one to two trophic levels higher than wild diets, and were less depleted in δ^13^C, two to four trophic levels higher than their natural dietary source.

Laundered echidnas are likely to spend some time in captivity prior to being traded. Although no information is available on growth rates of quills, we showed that elemental signatures were robust to detecting wild-caught echidnas when individuals had been in captivity for at least 6 months. Testing of a wild-captive quill (W-C1) suggested that while the isotopic signature had potentially shifted towards the captive group (Fig. 2), elemental analysis clearly identified the quill sample as wild with only 4% of scan samples supporting misclassification. The benefit of using elemental analysis of keratin samples is that they are long-term records incorporating locational information over months or even years. This means that wild animals would need to be kept in captivity for long time periods before their elemental signature shifted to captive.

Our findings are significant as they provide strong support for x-ray fluorescence as a new forensic tool for combating the global illegal wildlife trade. It should be noted that our study was limited in both sample size and spatial extent, while our captive echidnas were from the same source and fed the same diet. Further research is needed to ascertain detection accuracy among a more diverse set of samples, as well as applying the technique to other highly traded species with keratinous tissues such as birds (feathers), reptiles (scutes, nails, sloughs), and mammals (nails, scales, quills). An advantage of this technique is that it is non-invasive: samples can be taken from animals with minimal handling, and sometimes no handling, while repeat scans can be taken from samples to reduce sensitivity to outliers while still maintaining the ability to examine phenological patterns or post-treatment differences. Here we employed high resolution XRF in a laboratory setting to detect differences between captive and wild echidna quills, however, the method has great potential as an *in situ* forensic tool as hand-held multi-elemental XRF equipment are available. While hand-held devices typically scan for only a handful of elements at a time, machine learning provides a robust technique for obtaining sufficient predictive accuracy when combined with high resolution training sets. Our research team are currently measuring elemental abundances using hand-held devices to distinguish between captive and wild animals to provide an economical field-based tool for use in places like markets and farms and during border control where trading of animals frequently occurs.

## Methods

### Sample collection and preparation

Quills from captive short-beaked echidnas were sourced from Taronga Zoo, Taronga Conservation Society Australia (Sydney, Australia). Quills from wild echidnas were sourced from those presented for veterinary care or post-mortem examination at the Taronga Wildlife Hospital at Taronga Zoo and from the Wildlife Veterinary Department of Mogo Zoo (Mogo, Australia). A total of 24 quills were sampled from 22 echidnas. Twelve captive samples were collected from adult residents at Taronga Zoo for between 22 months and 20 years ($\bar{x}$ = 10.5 years). All captive echidnas were fed the same diet. Eleven wild quill samples were collected from ten individuals presented for veterinary care. One wild echidna was sampled twice after 1 and 7 days in care. Echidnas classified as wild had quills sampled at between 0 and 26 days in care ($\bar{x}$ = 4 days). These echidnas had been recovered or rescued from a range of locations within 290 km of Sydney, Australia. One echidna was classified as wild-captive as it was sampled 188 days after being kept for veterinary care. Quills were cleaned of any surface contaminants using a lint free cloth and deionized water and stored in paper envelopes at room temperature. A sample of Taronga Zoo captive echidna diet (Vetafarm, Wagga Wagga, 2700, NSW, Australia) was oven dried for 48 hours at 60 °C, homogenised using a mortar and pestle, and ground to a fine powder for stable isotope analysis.

All methods were carried out in accordance with approvals authorised by the Taronga Conservation Society Animal Ethics Committee (3a/02/15, 4a/02/18 and R16B229).

### Elemental and SIA analyses

Elemental analysis was performed at the Australian Nuclear Science and Technology Organisation (ANSTO) Lucas Heights facility using an ITRAX micro X-ray fluorescence (μXRF) core scanner. Whole quills were mounted length-ways along the ITRAX scanning platform and scanned at a resolution of 200 μm. Quills were aligned using a laser beam to ensure the scanning path (4 mm wide) covered all quills. A Molybdenum target tube was used with a dwell time of 10 seconds at settings of 30 kV and 55 mA. Elemental abundance data was collected for 24 elements (Fig. 1). Following ITRAX scanning the same quills were then ground using a ball mill (Retsch Mixer Mill 200) to create a homogenized powder for each quill. Samples were weighed into tin caps and stable carbon (^13^C) and nitrogen (^15^N) isotopes were analysed at the Bioanalytical Mass Spectrometry Facility (BMSF) at the Mark Wainwright Analytical Centre (MWAC) University of New South Wales. A Delta V Advantage Isotope Ratio Mass Spectrometer and Flash 2000 Organic Elemental Analyzer fitted with a Conflo 4 was used. A sample of captive diet from Taronga Zoo was also analysed for ^13^C and ^15^N for comparison with captive quill results. Data are available upon request from the authors.

### Statistical analyses

Differences in elemental signatures between captive-bred and wild-caught echidnas were tested by constructing univariate generalised mixed models in the ‘lme4’ package in R^43^. Negative binomial models were used to account for overdispersed data, while quill identity was used as a random variable to account for nesting of scans. We then used the tree-based machine learning algorithm random forest^44^ to determine whether quill samples from echidnas could be classified using elemental composition and δ^13^C and δ^15^N isotope ratios, implemented in the ‘randomForest’ package in R. Random forest is a machine learning technique that constructs an ensemble of unpruned classification trees by bootstrapping samples of training data and random predictor variables for tree induction^44^. Classification is made by aggregating (majority vote or averaging) the predictions of the ensemble. Two sets of random forest models were built for elemental and stable isotope data separately, running the default number of 500 trees and four variables at each split for elemental data and one variable for isotopic data. Variable importance was determined by ranking the mean decrease in accuracy for each predictor variable and by examining partial dependency plots (Supplementary Information).

### Ethics

This work was carried out under Animal Care and Ethics approvals from Taronga Conservation Society: AEC 4a/02/18 (previously prior to Feb 2018 it was 3a/02/15). Title: *Collection of opportunistic samples for researchers from live animals during veterinary procedures or routine husbandry procedures*. And Taronga Conservation Society Internal Sampling Licence Approval number R16B229. Title*: Tracing the trade: using keratinous tissue to combat illegal wildlife trade*.

## Electronic supplementary material

Supplementary Information

## Data Availability

The datasets generated during and/or analysed during the current study are available from the corresponding author on reasonable request.
