# Supplementary material for: Novel detection of provenance in the illegal wildlife trade using elemental data
Source: Sci Rep. 2018 Oct 18;8:15380. doi: 10.1038/s41598-018-33786-0 (PMC6194005; doi:10.1038/s41598-018-33786-0)

Supplementary Information

Novel detection of provenance in the illegal wildlife trade using elemental data

Kate J. Brandis, Phoebe J.B. Meagher, Lydia J. Tong, Michelle Shaw, Debashish Mazumder, Patricia Gadd & Daniel Ramp

1. Partial dependency plots for A) elemental data and B) isotopic data.

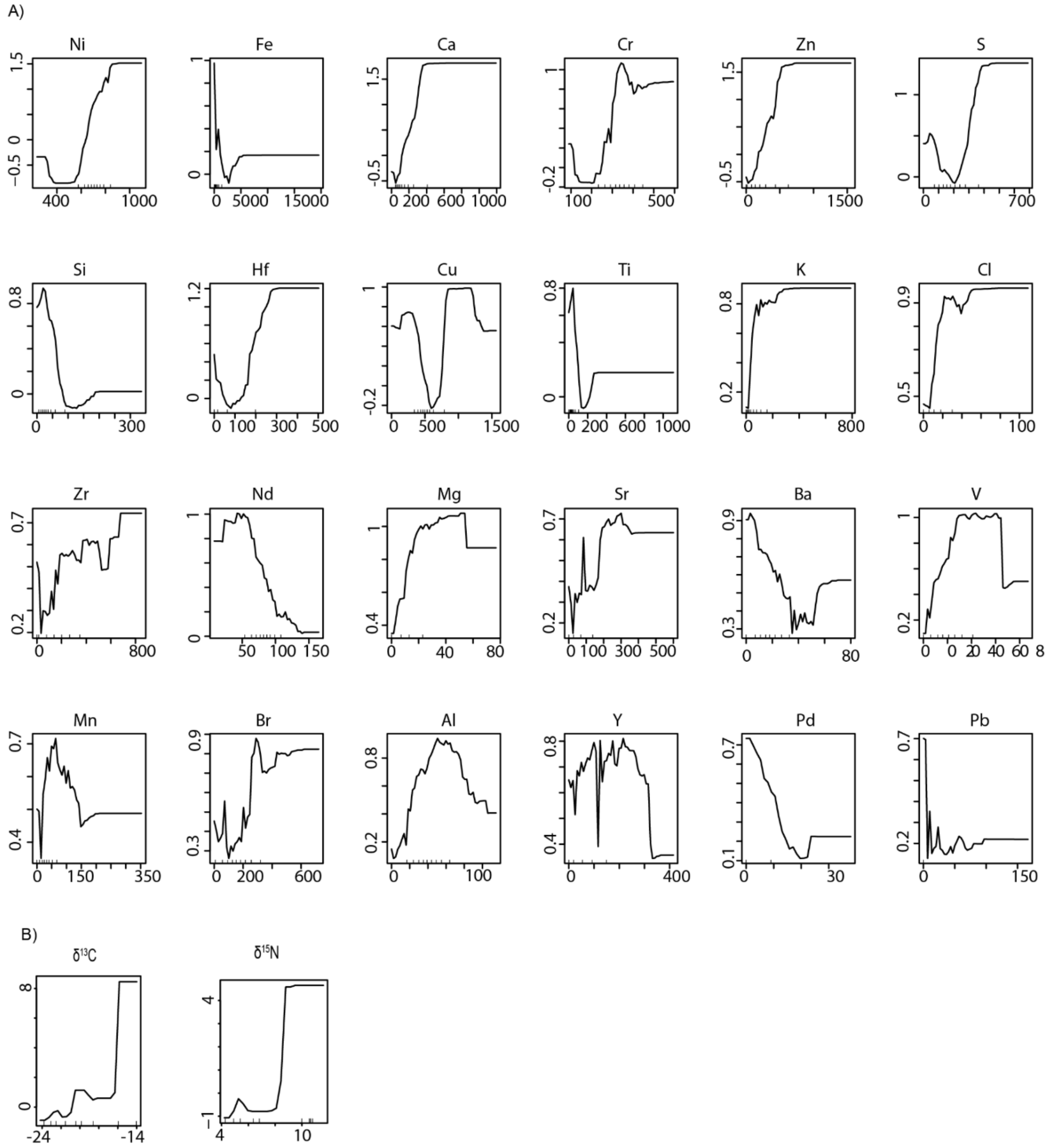

Supplement: Supplementary file 1 — Supplementary Information [file 41598_2018_33786_MOESM1_ESM.pdf]
